# Adherence to COVID-19 Appropriate Behavior Among Respiratory Therapists: A Cross-Sectional Study in the Kingdom of Saudi Arabia

**DOI:** 10.3389/fpubh.2021.715982

**Published:** 2021-08-18

**Authors:** Noor Al Khathlan, Bijaya Kumar Padhi

**Affiliations:** ^1^Department of Respiratory Care, College of Applied Medical Sciences, Imam Abdulrahman Bin Faisal University, Dammam, Saudi Arabia; ^2^Department of Community Medicine & School of Public Health, Post Graduate Institute of Medical Education and Research, Chandigarh, India

**Keywords:** respiratory care, respiratory therapist, COVID-19 protective behavior, transmission-reducing behaviors, Saudi Arabia, COVID-19 appropriate behavior

## Abstract

**Background:** Adherence to novel coronavirus disease 2019 (COVID-19) appropriate behavior plays a crucial element in the management of the infections of COVID-19. Despite the importance of transmission-reducing behaviors among healthcare professionals, there is a lack of literature in this area of research explicitly relating to respiratory therapists (RTs). Therefore, it is essential to assess the adherence level to COVID-19 transmission-reducing behaviors among the RTs in Saudi Arabia.

**Methods:** A web-based online survey was conducted using questions based on the risk assessment guidelines of WHO. A random representative sample of RTs (*N* = 215) residing in Saudi Arabia was recruited for the study. Descriptive and inferential statistics were computed using STATA software. Logistic regression analysis was used to identify key factors that are associated with adherence to COVID-19 appropriate behavior among the study participants.

**Results:** Of the 215 participants, 59.5% were aged between 26 and 35 years, and 40.9% were women. Most (85.5%) participants had a bachelor's degree while 12.0% had a master's degree. About 56.2% of RTs provided direct care to a confirmed patient of COVID-19 during the study periods. The study showed 80.9% of RTs in Saudi Arabia adhered to personal protective equipment (PPE) at the workplace and 65.0% at home. Moreover, the findings of the study indicated that senior RTs (with >5 years of experience) demonstrated a higher adherence level to the guidelines than RTs with <5 years of experience. High-risk perception [aOR:2.32; 95% CI: 1.09–3.27], and work history of <5 years [aOR:2.00; 95% CI: 1.14–3.15], were found to be the strongest predictors in explaining the adherence to appropriate behavior among the RTs at the workplace. Whereas the high-risk perception [aOR:2.32; 95% CI: 1.09–3.27] and being married [aOR:1.85; 95% CI: 1.08–3.82] were found to be the strongest predictors at home.

**Conclusion:** Adherence (“Always” or “Most times”) to COVID-19 appropriate behavior was found to be high at hospital settings among the study participants. However, the same practice was found to be inconsistence in non-healthcare settings among the RTs. Considering the paramount role of COVID-19 appropriate behavior in reducing the transmissions the policy focus, therefore, needs to be on creating a well-spread behavior change communication that is curtailing the adoption of appropriate behavior in the non-healthcare settings.

## Background

The novel coronavirus disease 2019 (COVID-19) has left the world reeling, the effects on the public health systems across the world has been devastating. It has posed significant challenges for the healthcare workers, and their safety thus must be ensured. Respiratory care (RC) is an important medical specialty which deals with the diagnosis and treatment of patients with acute and chronic respiratory diseases. Respiratory therapists (RTs) work alongside doctors in the various areas such as intensive care units (ICUs), emergency departments, out-patient departments, and home care settings. Involvement of RT in caring patients with COVID-19 is found to be extremely vital at this juncture (1, 2). Their expertise in assessing blood oxygen levels, assisting with intubations and bronchoscopies, and specializing in management of ventilator has been extremely valuable, and sometimes exposed them to the sickest patients and to dangerous aerosolizing procedures. In view of the importance of the specialty of RC and the effective role of an RT, public and private universities in the Kingdom have begun to offer RC programs since the 1988 (3). Though the RC profession itself has been recognized in Saudi Arabia over 40 years, but still not fully appreciated in the Saudi hospitals (3). Currently, around 10 universities offer RC programs. It has been reported that out of 411 hospitals in the country, only 88 hospitals offer services by the trained RTs, which represent 21.4% of total number of working hospitals in the country (4). As of December 2020, the total number of active RTs in Saudi Arabia is 1,477, one RT for every 17,629 population of Saudi (4). Half of them, work in the central part of the country and one quarter are Saudi nationals (4). Furthermore, majority of the RT workforce in the Kingdom, work in critical care settings which represents about 44.5% (4).

Numerous studies have reported inadequate training on infection prevention and control, and shortages of personal protective equipment (PPE) resulting in high rates of COVID-19 among healthcare workers (5, 6). In China, a study reported inadequate PPE as a reason for infection among healthcare workers (7).A systematic reviews and meta-analysis estimated that 21.7% of the healthcare workers experienced depression, 21.5% PTSD, and 22.1% anxiety during the pandemic (8). The Middle East reported the highest pooled rates of depression (34.6%) and anxiety (28.9 %) (8).

As RTs continue to run the ventilators that are keeping many patients with COVID-19 alive, majority of procedures that therapists perform occur at the head of the bed of the patients, putting therapists at a high risk for infection. Adherence to COVID-19 appropriate behavior plays a crucial element in the management of the infections of COVID-19. Despite the importance of adherence to the guidelines of COVID-19, there is a lack of literature in this field of research specifically relating to RTs. However, in the last year, RTs have been playing a major role in treating patients hospitalized with COVID-19. RTs have often been overlooked during the COVID-19 epidemic, even though they have played a crucial role. Therefore, it is essential to assess the risk perception and adherence level to the guidelines of COVID-19 among RTs in Saudi Arabia. The study was planned to assess the risk perception and adherence level of RTs to the guidelines of COVID-19 in Saudi Arabia.

## Methods

### Ethics Consideration

Ethical approval was obtained from Deanship of Scientific Research (DSR) at Imam Abdulrahman Bin Faisal University (IAU), Dammam, Saudi Arabia. Informed consent was provided to all the survey participants prior to their enrolment. Participants were allowed to terminate the survey at any time they desire. The survey data analysis and reporting ensured compliance with ethical requirements of Imam Abdulrahman Bin Faisal University (IAU), Dammam, Saudi Arabia.

### Study Design and Sample

The study was a cross-sectional, hospital-based survey conducted via simple random sampling. The study was conducted between March and June 2020 (the first phase of pandemic). RTs (*N* = 215) were randomly sampled from the list of RTs (as of December 2019) in the country and were asked to participate in this study. We collected the email address, and phone numbers of RTs working in various departments of RC across the country. The survey link was shared to them those who consented to participate in the survey. The target sample size of the participants was determined using the formula, *N* = Zα2P(1 – P) / d2, in which α = 0.05 and Zα = 1.96, and the estimated acceptable margin of error for proportion d was 5%. The proportion of RTs with COVID-19 appropriate behaviors was estimated at 80%, based on the available literature on infection control behaviors among healthcare professionals (9–11). The sample size was calculated using OpenEpi software (v3.01).

### Development of Study Questionnaires

A draft version of the survey was developed by the research team based on questionnaires of the WHO risk assessment guideline (12), and constructs of health predictive models (13–16). The draft questionnaires were validated and tested measures where possible, and then shared with the expert for their opinion. The draft survey was modified based on the feedback received to ensure question wording was clear and easily understood. A bilingual survey (Arabic and English) was shared to study participants to record their response.

### Outcomes and Covariates

The study focused on adherence to COVID-19 appropriate behaviors that reduce transmission of COVID-19. Adherence to each behavior was assessed using a 5-point response scale (always, most times, sometimes, rarely, never) in two occasions (hospital duty and non-hospital settings). Respondents were asked to record the extent to which they had adhered to each behavior over the previous week. The covariates, sociodemographic characteristics such as age, gender, nationality, hospital type (private vs. government), geographical region, and education status were assessed. Risk perception was assessed using a three-point response scale “Very Concerned” (High-risk perception); “Somewhat Concerned” (Moderate-risk perception); and “Not at all Concerned” (Low-risk perception) to the question “How concerned are you that you or someone in your family will be infected with COVID-19 virus?”

### Statistical Analysis

The anonymized data were further categorized, sex (male or female), age (18–25, 26–30, 31–40, or >40 years), marital status (married or single), educational level ( ≤ undergraduate or ≥postgraduate), technical title (junior, intermediate, or senior), geographic location (East, West, North, South, and Central province), nationality (Saudi or foreign), and type of hospital (secondary or tertiary). RTs were asked to record whether they were directly engaged in the clinical activities or providing care to patients with COVID-19. A composite variable “adherence to COVID-19 appropriate behaviors” was constructed by dichotomizing all key variables mentioned in Tables 1, 2. From the Likert response questions, the top two responses (e.g. “always” and “most times”) received a “1” and the three other responses (e.g., “sometimes,” “rarely,” or “never”) received a “0”.

**Table 1:** Adherence to COVID-19 appropriate behavior at workplace (*N* = 215).

**Variables**	***n* (%)**
Wear personal protective equipment (PPE)?	
Always as recommended	174 (80.93%)
Most of the times	9 (4.19%)
Occasionally	32 (14.88%)
Single gloves	
Always as recommended	161 (74.88%)
Most of the times	10 (4.65%)
Occasionally	5 (2.33%)
Rarely	39 (18.14%)
Medical mask	
Always as recommended	173 (80.47%)
Most of the times	11 (5.12%)
Occasionally	4 (1.86%)
Rarely	27 (12.56%)
Face shield or goggles/protective glasses	
Always as recommended	135 (62.79%)
Most of the times	31 (14.42%)
Occasionally	12 (5.58%)
Rarely	37 (17.21%)
Disposable Gown	
Always as recommended	169 (78.60%)
Most of the times	15 (6.98%)
Occasionally	2 (0.93%)
Rarely	29 (13.49%)
Remove and replace PPE according to protocol*	
Always as recommended	149 (69.30%)
Most of the times	27 (12.56%)
Occasionally	1 (0.47%)
Rarely	38 (17.67%)
Before and after touching the COVID-19 patient?	
Always as recommended	178 (82.79%)
Most of the times	9 (4.19%)
Rarely	28 (13.02%)
Before and after any clean or aseptic procedure	
Always as recommended	180 (83.72%)
Most of the times	7 (3.26%)
Occasionally	2 (0.93%)
Rarely	26 (12.09%)
After exposure to body fluid?	
Always as recommended	177 (82.33%)
Most of the times	10 (4.65%)
Occasionally	2 (0.93%)
Rarely	26 (12.09%)
After touching the COVID-19 patient's surroundings	
Always as recommended	171 (79.53%)
Most of the times	15 (6.98%)
Occasionally	3 (1.40%)
Rarely	26 (12.09%)

**Table 2:** Adherence to COVID-19 appropriate behavior other than healthcare setting (*N* = 215).

**Variables**	***n* (%)**
Disinfect home	
Always as recommended	118 (54.88%)
Most of the times	58 (26.98%)
Occasionally	31 (14.42%)
Rarely	8 (3.72%)
Wash hands frequently with soap or hand sanitizer	
Always as recommended	169 (78.60%)
Most of the times	40 (18.60%)
Occasionally	6 (2.79%)
Cover mouth and nose when coughing or sneezing	
Always as recommended	177 (82.33%)
Most of the times	32 (14.88%)
Occasionally	5 (2.33%)
Rarely	1 (0.47%)
Wear mask	
Always as recommended	102 (47.44%)
Most of the times	54 (25.12%)
Occasionally	37 (17.21%)
Rarely	22 (10.23%)
Avoided public transportation	
Always as recommended	172 (80.00%)
Most of the times	32 (14.88%)
Occasionally	7 (3.26%)
Rarely	4 (1.86%)
Avoided social activities	
Always as recommended	149 (69.30%)
Most of the times	51 (23.72%)
Occasionally	8 (3.72%)
Rarely	7 (3.26%)
Avoided going to hospital/clinic	
Always as recommended	114 (53.02%)
Most of the times	40 (18.60%)
Occasionally	18 (8.37%)
Rarely	43 (20.00%)
Avoided contact with people who have recently had a travel history	
Always as recommended	156 (72.56%)
Most of the times	47 (21.86%)
Occasionally	8 (3.72%)
Rarely	4 (1.86%)
Avoided going to crowded places	
Always as recommended	143 (66.51%)
Most of the times	47 (21.86%)
Occasionally	12 (5.58%)
Rarely	13 (6.05%)
Avoided contact with people who had fever or symptoms of respiratory illness	
Always as recommended	175 (81.40%)
Most of the times	24 (11.16%)
Occasionally	10 (4.65%)
Rarely	6 (2.79%)
Avoided Shaking Hands	
Always as recommended	180 (83.72%)
Most of the times	16 (7.44%)
Occasionally	14 (6.51%)
Rarely	5 (2.33%)

To determine potential predictive factors for adherence to COVID-19 appropriate behavior among participants, multivariable logistic regression analysis was performed. Model estimates were presented as ORs and 95% CIs, after adjustment for confounders, including sex, age, marital status, educational level, technical title, place of residence, and type of hospital. Data analysis was performed using STATA statistical software version 15.0 (STATA Corp.). The significance level was set at α = 0.05, and all tests were two-tailed.

## Results

A total of 215 RC professionals participated in this study. Of the 215 participants, 59.5% were aged 26–35 years, 40.9% were female and most of them had completed bachelor's degree. About 20% were of non-Saudi nationality, and half of them were having work history of more than 5 years. Approximately 56.2% provided direct care to a confirmed COVID-19 patient, and about 47.9% reported in engaging aerosol-generating procedures (AGP). About half of them were from government hospital under Ministry of Health, and 54.8% belonged to the eastern provinces of the country (Table 3).

**Table 3:** Socio-demographic characteristics, and risk perception to COVID-19 of the study participants (*N* = 215).

**Variables**	***n* (%)**
Age (Years)	
18–25	34 (15.81%)
26–35	128 (59.53%)
Above 35	53 (24.65%)
Gender
Male	127 (59.07%)
Female	88 (40.93%)
Highest education
PhD	5 (2.33%)
Masters	26 (12.09%)
Bachelor	184 (85.58%)
Nationality
Saudi	173 (80.47%)
Non-Saudi	42 (19.53%)
Years of working experience as RT
>10 Years	65 (30.23%)
6–10 Years	40 (18.60%)
1–5 Years	86 (40.00%)
<1 Year	24 (11.16%)
Type of hospital
MOH-Governmental hospital	105 (48.84%)
Non-MOH Governmental hospital	83 (38.60%)
Private hospital	27 (12.56%)
Hospital geographical location
Eastern Provinces	118 (54.88%)
Western Provinces	38 (17.67%)
Southern Provinces	8 (3.72%)
Central Provinces	51 (23.72%)
Direct care to a confirmed COVID-19 patient
No	94 (43.72%)
Yes	121 (56.28%)
Present when any aerosol generating procedures (AGP)
No	112 (52.09%)
Yes	103 (47.91%)
Risk Perception
Low	41 (19.06%)
Moderate	60 (27.90%)
High	114 (53.02%)

Table 1 presents participants adherence to COVID-19 appropriate behavior at workplace. Most (80.9%) of the study participants reported that they always wear PPE as recommended. Of the 215 respondents, 74.8% always used single gloves, 80.4% always used medical mask, 62.7% always used face shield, 62.7% always used disposable gown. About two-third of the study participants reported that they removed and replaced PPE according to protocol (Table 1).

Table 2 shows participants adherence to COVID-19 appropriate behavior other than healthcare setting. Of the 215 respondents, 54.8% always disinfect home, 78.6% wash hands frequently with soap or hand sanitizer, and 82.3% cover mouth and nose when coughing or sneezing. Only 47.4% reported that they wear mask at home. Most of them (80.0%) avoided public transportation, and 69.3% social activities. About half of the study participants reported that they prefer not to visit hospital/clinic during the pandemic. Approximately 72.5% reported that they will prefer to avoid contact with people who recently had a travel history, and 66.5% avoided going to crowded places. Approximately 81.4% avoided contact with people who had fever or symptoms of respiratory illness, and 83.7% avoided shaking hands (Table 2).

Table 4 depicts the logistic regression analyses for association between protective behaviors and socio-demographic characteristics of the study participants. The multivariable logistic regression model indicated that risk perception of participants to COVID-19 infection was significantly predicting the protective behavior at workplace (aOR = 2.32, *p* < 0.01), and non-workplace settings (aOR= 2.81, *p* < 0.01). Participants who were married had 1.68 times more likely to practice protective behavior at home (aOR= 1.68, *p* < 0.01). Similarly, participants who had a work history of 5 years and more were found to be adherence to the recommended protective behavior in both the settings (Table 4).

**Table 4:** Logistic regression analyses for association between appropriate behaviors and socio-demographic characteristics of the study participants (*N* = 215).

**Variables**	**“Adherence to COVID-19 appropriate behaviors”**			
	**At workplace**		**At home**	
	**OR [95% CI]**	**aOR [95% CI]**	**OR [95% CI]**	**aOR [95% CI]**
**Risk perceptions**
Low	Ref		Ref	
Moderate	1.38 [0.82–2.32]	1.35 [0.97–2.90]	1.25 [0.93–2.56]	1.22 [0.98–2.31]
High	2.56 [1.08–3.48] ^*^	2.32 [1.09–3.27] ^*^	2.65 [1.15–4.61]^*^	2.81 [1.13–4.50]^*^
**Age**
18–25	Ref		Ref	
Above 26	1.73 [0.91–2.96]	1.85 [0.98–3.63]	1.72 [0.91–3.10]	1.60 [0.97–3.41]
**Gender**
Male	Ref		Ref	
Female	1.13 [0.85–2.75]	1.20 [0.93–2.90]	1.27 [0.95-−2.84]	1.30 [0.98–2.68]
**Marital status**
Single	Ref		Ref	
Married	1.36 [1.08–2.41] ^*^	1.35 [1.07–2.30] ^*^	1.70 [1.03–3.17] ^*^	1.68 [1.06–2.95] ^*^
**Nationality**
Saudi	Ref		Ref	
Non-Saudi	1.27 [0.89–1.80]	1.35 [0.96–1.90]	1.17 [0.79–1.74]	1.35 [0.96–1.90]
**City of residence**
Central	Ref		Ref	
East	1.14 [0.66–1.33]	1.15 [0.99–2.00]	1.20 [0.80–2.13]	1.22 [0.99–2.15]
West	1.21 [0.79–2.55]	1.25 [0.78–2.33]	1.23 [0.79–1.92]	1.25 [0.66–2.43]
South	1.35 [0.96–2.40]	1.32 [0.79–2.46]	1.41 [0.90–2.00]	1.45 [0.91–2.20]
**Highest education**
Bachelors	Ref		Ref	
Masters and above	1.65 [0.96–2.42]	1.68 [0.94–2.51]	1.50 [0.86–2.78]	1.58 [0.93–2.75]
**Workplace**
Government	Ref		Ref	
Private	1.18 [0.74–2.46]	1.32 [0.90–2.47]	1.19 [0.70–2.60]	1.21 [0.78–2.85]
**Work history**
<5 years	Ref		Ref	
5 years and above	2.00 [1.13–3.80] ^*^	2.08 [1.14–3.15] ^*^	1.96 [1.21–3.70] ^*^	2.12 [1.10–3.20] ^*^
**Direct care**
No	Ref		Ref	
Yes	1.21 [0.89–2.93]	1.30 [0.91–2.83]	1.41 [0.85–2.80]	1.40 [0.90–2.92]

* *P <0.01*.

## Discussions

To the best of our knowledge this is the first study that provides evidence of adherence to precautionary behavioral practice toward COVID-19 among RTs in the Kingdom of Saudi Arabia and elsewhere. These healthcare professionals are found to be extremely valuable during the COVID-19 pandemic, and understanding their behavioral practices is extremely critical for policy implications. The findings of this study are vital for risk management and healthcare policy in the Kingdom and elsewhere. The study reported that most (80.9%) of the RC professionals followed recommended practice at workplace to the highest level. However, the appropriate behavioral practice toward COVID-19 at home or non-healthcare settings was found to be compromised (65.0%). The multivariable logistic regression analysis indicated a positive association between the higher risk perception toward the COVID-19 infection with adherence to precautionary behavior practices at both settings.

Since this is the first study in its nature, a direct comparison is limited. A qualitative comparison can be made with other groups of healthcare professionals. The risk perception and adherence level to the recommended preventive strategies among the study participants in this study was found to be much higher than the study reported elsewhere (17–20). A study conducted in China indicated that 89% of the healthcare providers are knowledgeable regarding preventive methods of COVID-19 (21). A study conducted in Ethiopia highlighted that there was a gap on recommended precautionary measures, especially for wearing masks and gloves among the healthcare professionals (17). The study reported that well-educated participants better exercise protective behavior (17).

Adherence to preventive measures for COVID-19 in Saudi Arabia among general population was found to be moderately high (22–24). A cross-sectional study conducted during the same periods to our study reported that participants aged 25–34 years were 25% less likely to comply with hand hygiene and social distancing (24).

The lower levels of adherence of COVID-19 appropriate behavior among the study participants in home environment needs further investigation, may be attributable to the poor habit of PPE wearing, a perception that they interfere with performing tasks. The scientific understanding of precautionary behavioral practice of among RTs is beyond the scope of the current study.

The key strengths of this study are that we used an appropriate random sample of participants across the country; the outcome variables were measured using a Likert scale than dichotomized response (Yes/No); and the study reported the behavioral practices at both settings—home, and workplace of the study participants. Some limitations to consider in the study are we relied on self-reported data of the participants who might be influenced by their RC specialty to self-report applications of high-protective measures. We have not accounted some vital information such as history of infection and vaccination among them or their families or coworkers, which potentially affect the behaviors of the participants. Next, discussing theories that could explain the behaviors beyond scope of the paper. The reported behavior could vary overtime, since the data were collected during the first wave of the pandemic, it will be interesting to study the change of behavior with the progress of the pandemic. In spite of the aforementioned limitations, the study has several advantages and warrants further investigation of this unique healthcare professional.

## Conclusion

The study findings present a concerning outlook for the RT, a group continually needed at the forefront against COVID-19. The adherence level to recommended precautionary behavioral practice toward COVID-19 among the participants on the study was found to be much higher. Although most the participants reported recommended protective measures to the highest level at workplace, there was a gap on adherence to the appropriate measures at non-workplace, especially, behavioral practice for wearing masks and disinfecting homes. Furthermore, the finding also indicated a statistically significant positive association between the risk perception and adherence of the participants to recommended behavioral practices. Therefore, there is a need to educate the importance of precautionary measures in the non-healthcare settings.

## Data Availability Statement

The raw data supporting the conclusions of this article will be made available by the authors, without undue reservation.

## Ethics Statement

Ethical approval was obtained from The Deanship of Scientific Research (DSR) at Imam Abdulrahman Bin Faisal University (IAU), Dammam, Saudi Arabia. All participants provided their written informed consent to participate in this study.

## Author Contributions

NA and BP conceptualized the study. NA conducted research, provided research materials, collected and organized data, as well as contributed substantially to writing the manuscript. BP analyzed and interpreted data and wrote the first draft of the article. All the authors have critically reviewed and approved the final draft and are responsible for the content and similarity index of the manuscript.

## Conflict of Interest

The authors declare that the research was conducted in the absence of any commercial or financial relationships that could be construed as a potential conflict of interest.

## Publisher's Note

All claims expressed in this article are solely those of the authors and do not necessarily represent those of their affiliated organizations, or those of the publisher, the editors and the reviewers. Any product that may be evaluated in this article, or claim that may be made by its manufacturer, is not guaranteed or endorsed by the publisher.
